# Research progress on cuproptosis in cancer

**DOI:** 10.3389/fphar.2024.1290592

**Published:** 2024-01-31

**Authors:** Qingbo Feng, Chenyu Huo, Maijian Wang, Handong Huang, Xingbin Zheng, Ming Xie

**Affiliations:** ^1^ Department of General Surgery, Digestive Disease Hospital, Affiliated Hospital of Zunyi Medical University, Zunyi, Guizhou, China; ^2^ West China School of Medicine, West China Hospital, Sichuan University, Chengdu, Sichuan, China

**Keywords:** cuproptosis, cancer, mechanism, therapy, review

## Abstract

Cuproptosis is a recently discovered form of cell death that is mediated by copper (Cu) and is a non-apoptotic form of cell death related to oligomerization of lipoylated proteins and loss of Fe-S protein clusters. Since its discovery, cuproptosis has been extensively studied by researchers for its mechanism and potential applications in the treatment of cancer. Therefore, this article reviews the specific mechanism of cuproptosis currently studied, as well as its principles and strategies for use in anti-cancer treatment, with the aim of providing a reference for cuproptosis-based cancer therapy.

## 1 Introduction

Cancer is a type of disease characterized by abnormal cell proliferation, and is one of the foremost reasons for mortality globally (Sung et al., 2021). Despite considerable progress in treatment, the overall cancer-related mortality rate has not significantly decreased. One of the key factors in cancer treatment is effectively killing cancer cells without harming non-malignant cells. Common methods used for cancer treatment, such as surgery, radiation therapy, chemotherapy, still have unavoidable adverse effects. Therefore, the search for more effective and tolerable cancer therapies continues (Mun et al., 2018; Wu et al., 2021). Programmed cell death (PCD) is necessary for maintaining cellular homeostasis and is a promising approach in cancer treatment. Apoptosis has long been considered the only form of cell death that can be targeted by pharmacological and genetic interventions for the development of anticancer drugs. However, innate or induced resistance of cancer cells to apoptosis often leads to fatal side effects and increased recurrence rates. Recent research has shown that there are many different forms of PCD that differ from the apoptosis mechanism, including necroptosis, ferroptosis, cuproptosis, NETosis, autosis, entosis, and parthanatos. These forms of PCD may overcome the limitations of apoptosis and provide new hope for cancer treatment (Holohan et al., 2013; Parisi et al., 2018).

Among the non-apoptotic forms of PCD, cuproptosis has gained widespread attention as a novel pathway for regulating cell death. It has long been discovered that the accumulated levels of Cu in the serum and tumor tissues of individuals with various malignant tumors, such as lung cancer (Jin et al., 2011), breast cancer (Pavithra et al., 2015), pancreatic cancer (Lener et al., 2016), thyroid cancer (Baltaci et al., 2017), colorectal cancer (Aubert et al., 2020), prostate cancer (Saleh et al., 2020), are significantly altered. Therefore, a series of copper-related studies have been conducted. Discovered and named by Tsvetkov et al., in 2022, cuproptosis is a cell death pathway triggered by copper. As early as 2019, Tsvetkov et al. identified a Cu-dependent form of cell death while studying the anticancer mechanism of elesclomol. They found through their research that elesclomol can exert effective anti-cancer activity by increasing reactive oxygen species (ROS) levels. Previous reports suggested that the Cu(II)-elesclomol complex was transported to the mitochondria where it was reduced to Cu(I), subsequently inducing ROS-dependent cell apoptosis (Nagai et al., 2012). However, the authors found that treatment with elesclomol resulted in cell death without activating caspase3, which is a classic hallmark of cell apoptosis (Elmore, 2007). Additionally, they exposed cells to inhibitors of established cell death pathways (caspase inhibitor for apoptosis, ferrostatin-1 for iron-dependent cell death, necrostatin-1 for necrotic cell death, and N-acetylcysteine for oxidative stress) after knocking out the key apoptotic factors BAX and BAK1. Nevertheless, despite these treatments, the researchers found that the cell death induced by Cu ionophores was not preventable. This indicates that the form of cell death induced by Cu is different from the known mechanisms of cell death. Further research carried out by the authors revealed that the mechanism may be that the elesclomol-Cu (II) complex may function as a new substrate for the reduced mitochondrial enzyme FDX1, resulting in oxidation and the generation of Cu (I), and promoting Cu-dependent cell death (Tsvetkov et al., 2019). Three years later, Tsvetkov and colleagues further elucidated the mechanism underlying the Cu-dependent cell death induced by elesclomol. Excess intracellular Cu (II) can be transported to the mitochondria via ion carriers, where FDX1 reduces Cu (II) to Cu (I). The direct binding of Cu (I) to lipoylated components of the tricarboxylic acid (TCA) cycle, particularly DLAT, induces the aggregation of lipoylated proteins and the loss of iron-sulfur cluster proteins, resulting in proteotoxic stress that eventually leads to cell death (Tsvetkov et al., 2022). More details will be elaborated on in detail later in the text.

To date, there has been a considerable amount of research on copper-induced cell death. Therefore, this article will review the current understanding of the specific mechanisms of cuproptosis and its therapeutic implications for cancer treatment, aiming to provide direction for future research related to copper-induced cell death and cancer.

## 2 Cu and cancer signaling pathway

**FIGURE 1:**
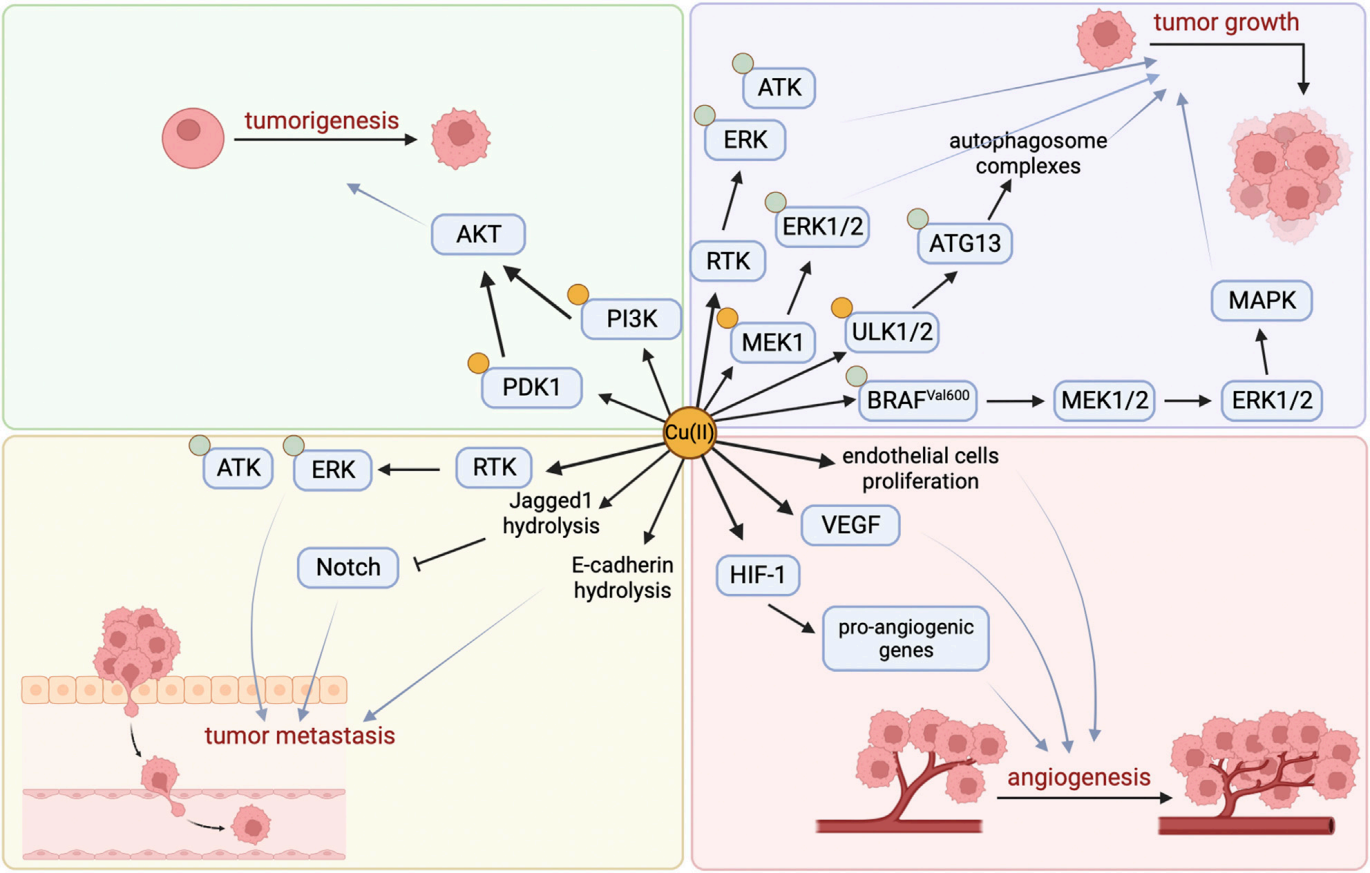
Cu-related Cancer signaling pathway.

The development of cancer is closely related to Cu, which activates related pathways by binding to key molecules in multiple signaling pathways in tumor cells, directly or indirectly affecting cancer. Studies have found that copper is essential for at least three features involved in cancer progression: cell proliferation, angiogenesis, and metastasis (Figure 1) (Li, 2020).

By binding and activating key molecules in multiple signaling pathways, copper is known to directly affect multiple signaling pathways in tumor cells (Table 1). Firstly, Cu (II) can significantly induce ligand-independent receptor tyrosine kinase (RTK) signaling pathways in cancer cells, and activated RTKs (including epidermal growth factor receptor (EGFR), hepatocyte growth factor receptor (MET), etc.) subsequently promote cancer cell migration and proliferation by causing downstream extracellular regulated protein kinases (ERK) and agammaglobulinemia tyrosine kinase (ATK) phosphorylation (He et al., 2019). Secondly, the promotion of tumor occurrence by Cu is also related to its activation of the phosphoinositide 3-kinase (PI3K)-protein kinase B (PKB, also known as AKT) signaling pathway. On the one hand, Cu can directly bind to PI3K to activate AKT (Ostrakhovitch et al., 2002), and on the other hand, it can activate downstream substrate AKT by binding to 3-phosphoinositide-dependent protein kinase 1 (PDK1), promoting tumor occurrence (Guo et al., 2021). Lately, it has been shown that Cu participates in the oncogenic BRAF signaling pathway in the mitogen-activated protein kinase (MAPK) pathway. BRAF^Val600^ phosphorylates and activates Mitogen-activated protein kinase kinase 1 (MEK1) and MEK2, which in turn phosphorylate and activate ERK1/2, stimulating the MAPK pathway and ultimately promoting tumor growth. In addition, Cu can directly bind with Cu-binding protein MEK1 to phosphorylate ERK1/2 and promote tumor growth (Brady et al., 2014). Besides, the autophagy pathway can recycle metabolic waste of tumor cells to ensure their energy supply or facilitate their evasion of apoptosis (Xie et al., 2023), which is beneficial for the growth of tumor cells. Cu is necessary for the activity of autophagy kinases ULK1 and ULK2, and it directly binds to them and acts as a regulatory factor to promote the phosphorylation and activation of autophagy-related protein 13 (ATG13), promote the formation of autophagosome complexes, and ultimately lead to tumor growth (Tsang et al., 2020; Xie et al., 2023).

**TABLE 1:** Cu-related Cancer signaling pathway.

Literature	Research samples	Function	Signaling pathway	Mechanism
Copper (II) Ions Activate Ligand-Independent Receptor Tyrosine Kinase (RTK) Signaling Pathway.	lung cancer, prostate cancer	promote tumor cell migration and proliferation	RTK signaling pathway	activated RTKs cause downstream ERK, ATK phosphorylation
1. Copper Ions Strongly Activate the Phosphoinositide-3-Kinase/Akt Pathway Independent of the Generation of Reactive Oxygen Species.2. Copper Promotes Tumorigenesis by Activating the PDK1-AKT Oncogenic Pathway in a Copper Transporter 1 Dependent Manner.	colon cancer	promote tumor occurence	PI3K-AKT signaling pathway	1. Cu can directly bind to PI3K to activate AKT2. activate downstream substrate AKT by binding to PDK1
Copper is required for oncogenic BRAF signalling and tumorigenesis.	melanoma	promote tumor growth	BRAF signaling pathway	1. BRAF^Val600^ phosphorylates and activates MEK1and MEK2, which in turn phosphorylate and activate ERK1/2.2. Cu can directly bind with MEK1 to phosphorylate ERK1/2.
Copper is an essential regulator of the autophagic kinases ULK1/2 to drive lung adenocarcinoma.	lung adenocarcinoma	promote tumor growth	autophagy pathway	Cu directly binds to ULK1/2 and acts as a regulatory factor to promote the phosphorylation and activation of ATG13, promote the formation of autophagosome complexes
1. Copper homeostasis as target of both consolidated and innovative strategies of anti-tumor therapy.2. The stimulation of angiogenesis and collagen deposition by copper.3. Targeting copper in cancer therapy: 'Copper That Cancer'.4. Role of copper in angiogenesis and its medicinal implications.	pathological cardiac hypertrophy	promote the formation of new blood vessels	HIF-1α	1. stimulate the proliferation and migration of endothelial cells 2. activate VEGF and other angiogenic factors3. stabilize HIF-1α to promote the expression of pro-angiogenic genes
Copper Modulates Zinc Metalloproteinase-Dependent Ectodomain Shedding of Key Signaling and Adhesion Proteins and Promotes the Invasion of Prostate Cancer Epithelial Cells	prostate cancer	Promote the metastasis of tumor cell	Notch signaling pathway	1. Cu can mediate the hydrolysis of Notch ligand---Jagged1 protein.2. Cu can promote the hydrolysis of E-cadherin.

Furthermore, Cu can induce many pro-angiogenic responses and is therefore considered a messenger that opens up the angiogenic pathway (Tisato et al., 2010). It can stimulate the proliferation and migration of endothelial cells (De Luca et al., 2019), activate vascular endothelial growth factor (VEGF) and other angiogenic factors (Gérard et al., 2010; Denoyer et al., 2015), stabilize hypoxia-inducible factor-1 (HIF-1) to promote the expression of pro-angiogenic genes (Xie and Kang, 2009), and ultimately promote the formation of new blood vessels.

Studies have shown that the Notch signaling pathway may play an important tumor suppressor role in certain tissues. Jagged1 belongs to the Notch ligand and Cu can mediate the hydrolysis of Jagged1 protein (Sethi and Kang, 2011; Parr-Sturgess et al., 2012; Nowell and Radtke, 2017). In addition, Cu can promote the hydrolysis of another key cell surface protein, E-cadherin, which is associated with cancer invasion and metastasis (Parr-Sturgess et al., 2012), indicating that Cu may be an important factor in promoting tumor cell metastasis.

## 3 Cuproptosis and cancer signaling pathway

Despite the aforementioned explanations, Cu can promote cancer development through a series of signaling pathways. However, researchers have also discovered that excessive Cu can induce tumor cell death.

**FIGURE 2:**
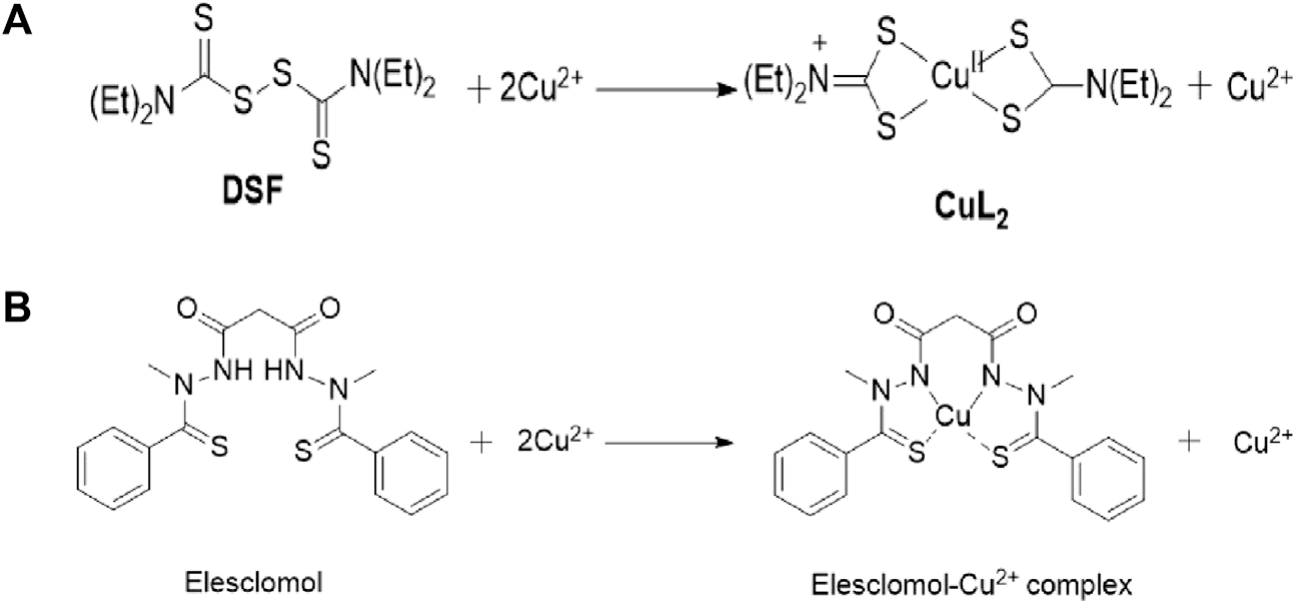
Chemical structure of DSF and Elesclomol and the proposed mechanism them bind to Cu.

As early as the 1980s, it was discovered that Cu could induce cell death (Halliwell and Gutteridge, 1984), but the mechanism was not elucidated at that time. We have already discovered through research that the Cu homeostasis mainly depends on Cu transport proteins SLC31A1 and ATP7A/B. SLC31A1 is responsible for the uptake of Cu, while ATP7A and ATP7B are responsible for the efflux of Cu (Migocka, 2015; Li et al., 2018). The mechanism of cell death caused by Cu homeostasis imbalance is consistent with that induced by Cu ionophores. Cu ionophores are lipophilic molecules that can reversibly bind to Cu ions and transport them through the cytoplasmic or mitochondrial membrane structure. As a novel type of anticancer drug (Oliveri, 2022), Cu ionophores have been crucial to the discovery of cuproptosis (Figure 2). Common Cu ionophores used in anticancer research include disulfiram (DSF), elesclomol (ES), etc. Many researchers believe that DSF and ES induce cell death through Cu, but the exact mechanism is not yet clear. Represented by the study of the ES-induced cell death mechanism, ROS is generated by mitochondria and can promote the activation of mitochondrial-dependent cell death pathways (Sabharwal and Schumacker, 2014). Many researchers have since generally believed that ES-induced cell death is due to an increase in ROS levels caused by various mitochondrial-related factors (Xie et al., 2023). In 2012, Nagai et al. found through the study of melanoma cell lines that ES transports Cu, which leads to a decrease in levels of mitochondrial-related proteins, resulting in an elevation in ROS levels and additional suppression of tumor cell proliferation (Nagai et al., 2012). In 2013, Yadava et al. found through their study in human leukemia K562 cells that Cu(II)-elesclomol can effectively oxidize ascorbic acid at physiological concentrations, producing harmful H2O2 through the reaction of Cu (I) with O2. The reaction of H2O2 with Cu (I) produces more destructive and highly active ROS (Yadav et al., 2013). In the 2015 study by Hasinoff et al. on ES, it was suggested that Cu-ES may have additional effects, such as blocking G1 phase cells to stop cell growth and inducing DNA double-strand breaks (Hasinoff et al., 2015). Other research on copper ionophores yielded similar mechanisms to those mentioned above.

In 2019, Tsvetkov et al. shed further light on the mechanism of action of ES. Their findings revealed that ES has a dual effect, inhibiting the function of FDX1, a crucial component in the assembly of Fe-S clusters, while also acting as a novel substrate to induce a distinct form of Cu-dependent cell death when bound to Cu. The researchers described the interaction between ES and the mitochondrial enzyme FDX1, which promotes the toxicity induced by ES through two different processes. Firstly, ES binds directly to reduced FDX1, thereby inhibiting its role in the biosynthesis of Fe-S clusters and serving as an upstream regulator of mitochondrial function. Secondly, the ES-Cu (II) complex acts as a new substrate for reduced FDX1, leading to oxidation and the production of Cu (I). This, in turn, triggers a unique form of Cu-dependent cell death that is not susceptible to inhibition by apoptosis or ferroptosis inhibitors (Tsvetkov et al., 2019). In 2021, it was discovered through a study of glioblastoma stem like cells (GSCs) that oxidative stress is the main mechanism by which ES acts on GSCs and GSC-derived endothelial cells (GdECs). Specifically, treating cells with ES can lead to alterations in mitochondrial membrane properties, an increase in mitochondrial ROS generation, and a reduction in glutathione (GSH) levels (Buccarelli et al., 2021), which are early molecular events indicating cell death (Matarrese et al., 2005).

Overall, in previous studies on the Cu-dependent form of cell death induced by ES, most researchers have summarized the mechanism of this form of cell death as Cu’s effect on mitochondria, leading to an increase in ROS production. Nevertheless, in a study on the mechanism of action of ES, the use of a 5 mM ROS inhibitor, N-acetylcysteine (NAC), could not eliminate the cytotoxicity induced by ES-Cu, and the use of 10 mM NAC could only partially eliminate the cytotoxicity of ES-Cu. Therefore, it is speculated that the increase in ROS is not the main cause of Cu-dependent cell death (Xie et al., 2023). Finally, in 2022, Tsvetkov et al. officially named the Cu-induced form of cell death as “cuproptosis” and refined its mechanism, marking a new stage in the investigation of Cu-induced cell death.

They found through their research that (15) the respiratory reserve capacity of cells was significantly reduced after treatment with copper ions, while the basal respiration or adenosine triphosphate (ATP)-related respiration remained relatively stable. The researchers also noted that cells that predominantly use mitochondrial respiration are approximately 1000 times more susceptible to Cu ion inducers than glycolytic cells. Treatment with mitochondrial antioxidants, fatty acids, and inhibitors of mitochondrial function all had a significant impact on cell sensitivity to Cu ions. Furthermore, inhibiting the electron transport chain (ETC) complex and blocking mitochondrial succinate uptake both decreased Cu-induced cell death. At the same time, the study found that the levels of metabolites related to the TCA in cells treated with Cu ion carriers changed.

**FIGURE 3:**
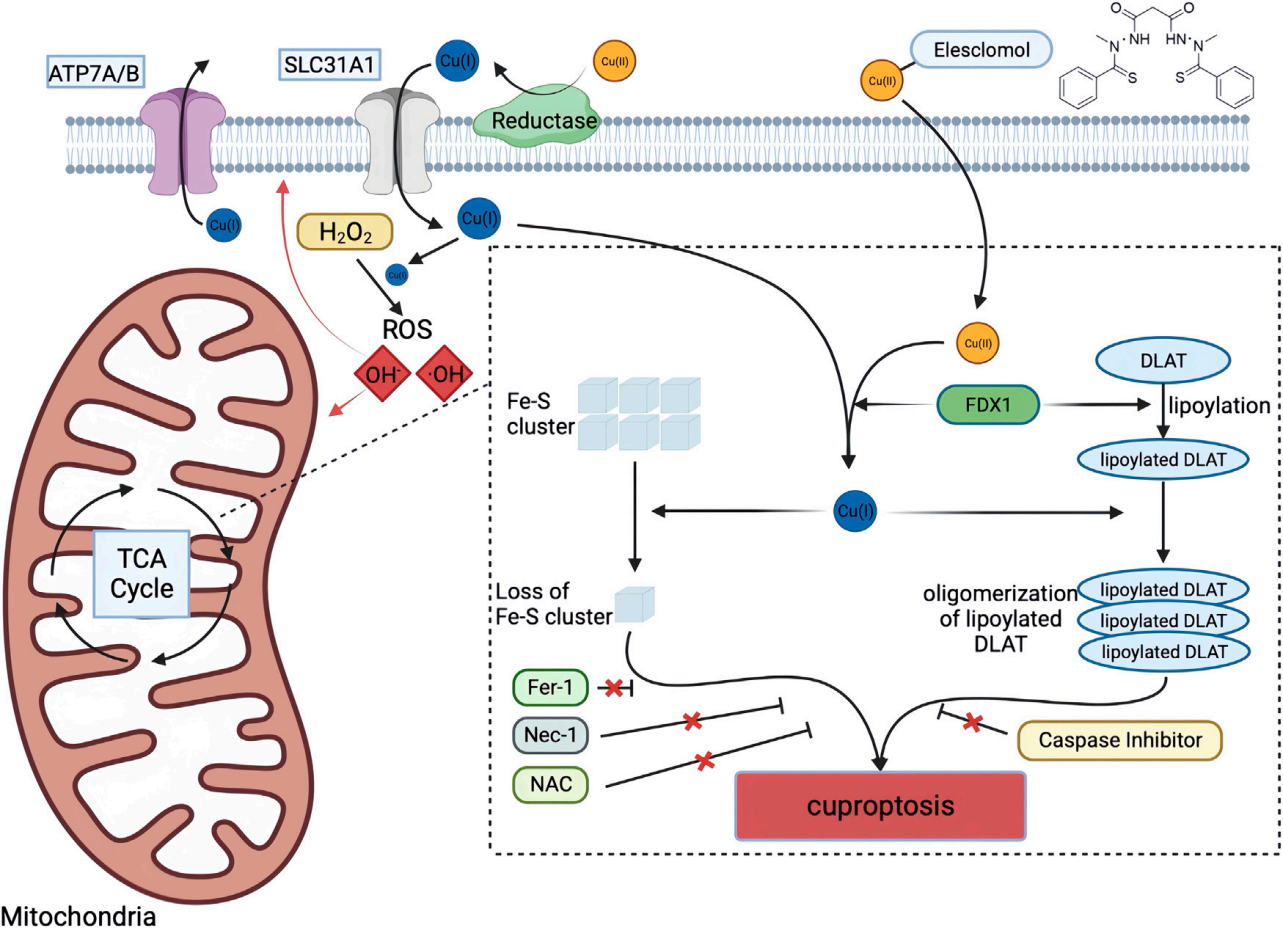
Schematic diagram of cuproptosis mechanism. Cu ionophores such as elesclomol bind extracellular Cu and transport it to intracellular compartments. Cu then binds to lipoylated mitochondrial enzymes in the TCA cycle such as DLAT, inducing the aggregation of these proteins. FDX1/LIAS is an upstream regulator of protein lipoylation, facilitating the aggregation of mitochondrial proteins and loss of Fe–S clusters. Together, these aberrant processes lead to proteotoxic stress and ultimately cell death. Created with BioRender. α‐KG α‐ketoglutarate, DLAT dihydrolipoamide S‐acetyltransferase, FDX1 ferredoxin‐1, Fe–S iron–sulfur, Fer‐1 ferrostatin‐1, LIAS lipoic acid synthetase, NAC N-acetylcysteine, Nec‐1 necrostatin‐1, TCA tricarboxylic acid.

These findings suggest that Cu-dependent cell death is dependent on mitochondrial respiration rather than ATP production, and that Cu does not directly target the ETC but rather components of the TCA cycle. The researchers used genome-wide CRISPR-Cas9 loss-of-function screens to identify genes involved in Cu ionophore-induced cell death. The results indicated that the loss of FDX1 and lipoate synthase (LIAS) provided resistance to Cu-dependent cell death, strengthening the relationship between FDX1, protein lipoylation, and Cu toxicity. Further investigation revealed that FDX1 is an upstream regulatory factor of protein lipoylation, and that Cu can directly bind and induce the oligomerization of lipoylated DLAT, leading to the loss of Fe-S cluster proteins. In summary, Cu-dependent cell death is mediated by a classical mechanism involving protein lipoylation, which occurs in only a few mammalian proteins mainly concentrated in the TCA cycle and is vital for enzymatic function (Mayr et al., 2014; Solmonson and DeBerardinis, 2018). When Cu ions accumulate inside the cell, they bind directly to the lipoylated components in the TCA cycle, causing protein aggregation and disruption. This ultimately blocks the TCA cycle, leading to protein toxicity stress and cell death. The study provides insight into the correlation between mitochondrial metabolism and the susceptibility of Cu-dependent cell death. Cells that rely more on mitochondrial respiration and are more active in the TCA cycle have a greater number of lipoylation enzymes, particularly the PDH complex. Lipoyl, a Cu binding moiety, can cause aggregation of lipoylated proteins, leading to the loss of Fe-S cluster proteins and the induction of HSP70 after Cu binding, indicating acute protein toxicity stress (Figure 3).

## 4 Cuproptosis and immune checkpoints

Clinical outcomes have been dramatically improved by blocking immune checkpoint signaling, particularly PD-1/PD-L1 signaling (Topalian et al., 2012).

It appears that intratumoral Cu levels affect PD-L1 expression within tumor cells. There is a strong association between CTR1 and PD-L1 across most cancer types, but not in matched healthy tissue (Voli et al., 2020). In tumor cells, Cu addition enhances PD-L1 expression and modulates immune escape induced by PD-L1. Conversely, Cu chelators (Dextran-catechin or TEPA) prevent STAT3 and EGFR phosphorylation and cause PD-L1 degradation via ubiquitination. In addition, Cu chelators increase the infiltration of CD8^+^ T cells and natural killer cells, which inhibits tumor growth. In spite of the fact that disulfiram and copper (DSF/Cu) fail to suppress tumor growth, disulfiram upregulates PD-L1 and inhibits T-cell infiltration by inhibiting PARP1 activity and improving GSK3 phosphorylation at Ser9 through the PARP1 gene (Zhou et al., 2019). Anti-PD-1 antibodies combined with DSF/Cu slow tumor growth even further. Tumor tissues contain copper ions, which Cu chelators can scavenge, thereby exerting their anticancer effects. An injectable Cu-induced hydrogel with anti-PD-L1 and nitric oxide (NO) enhances immunotherapy by amplifying immunogenic cell death and limiting cancer-associated fibroblasts (CAFs), which impedes immune cell infiltration as well (Shen et al., 2023).

**FIGURE 4:**
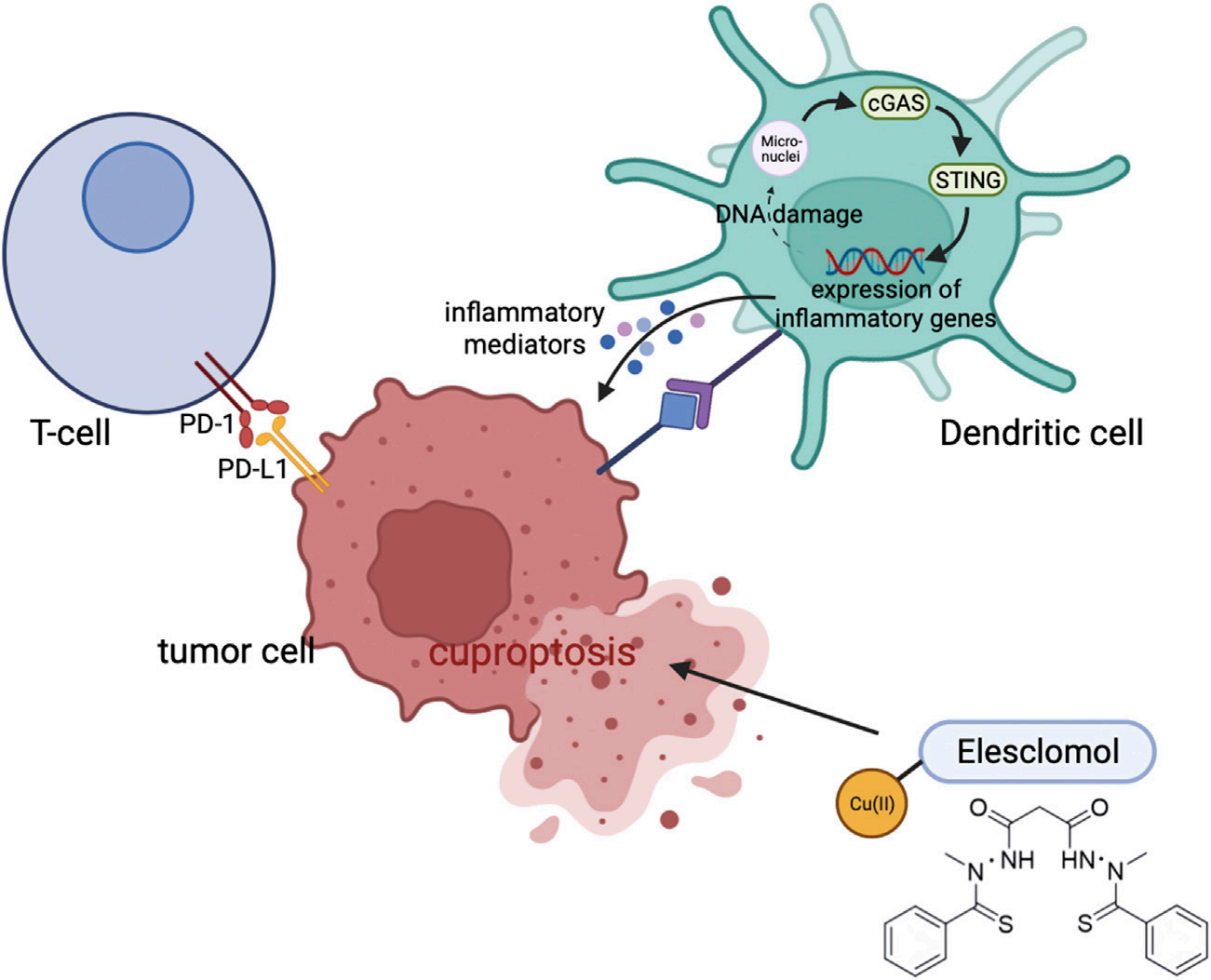
Cuproptosis can improve antitumor immunity through modulating the cGAS-STING signaling.

Uptake of Cu is mediated by the glycoprotein CD44 on the surface of the cell (Solier et al., 2023). An upregulation of CD44 leads to increased mitochondrial Cu2+ during macrophage activation. By catalyzing NAD(H) redox cycling, mitochondrial Cu2+ promotes metabolic changes and triggers epigenetic modifications that lead to inflammation. Moreover, DSF/Cu enhances antitumor immunity by triggering immune cell death, and also polarizes M1-macrophages and rewires glucose metabolism through mTOR (Zheng et al., 2020). Combining DSF/Cu and CD47 blockade enhances CD8^+^ T cell cytotoxicity by facilitating maturation of dendritic cells (Gao et al., 2022). Additionally, in clear cell renal cell carcinoma, cuproptosis improves cancer immunity by activating cGAS-STING signaling (Figure 4) (Jiang et al., 2022).

## 5 Cuproptosis and cancer treatment

Cu is an essential micronutrient for a wide variety of biological processes, such as mitochondrial respiration, antioxidant defense, and biosynthesis (Festa and Thiele, 2011). Importantly, the intracellular Cu concentration is kept at a relatively low range, and moderate increases can cause cytotoxicity and even lead to cell death. There are two types of copper ions in living organisms, cuprous ions (Cu+, reduced form) and copper ions (Cu2+, oxidized form). Homeostasis of Cu can be modulated in several ways, including Cu uptake, utilization, and export (Maung et al., 2021). Through ionophores, excess Cu2+ within cells is transported to mitochondria, where it is reduced to Cu + by FDX1. Cu + causes lipoylated proteins to aggregate and Fe-S cluster proteins to destabilize within the tricarboxylic acid (TCA) cycle, leading to cell death as a result of proteotoxic stress (Tsvetkov et al., 2022). Excessive copper can cause toxicity to different cells. Cu excess leads to imbalances of intracellular iron metabolism by disturbing assembly of iron-sulfur cofactors in *Bacillus subtilis* (Chillappagari et al., 2010). Cu targets the Fe-S domain by Yah1 in yeast and the Fe-S clusters of dehydratases are primary intracellular targets of Cu toxicity in *Escherichia coli* (Macomber and Imlay, 2009; Vallières et al., 2017).

Several copper chelators have been used to reduce copper bioavailability, including D-penicillamine, tetrathiomolybdate (TM), and tetraethylenepentamine (TEPA) (Liu et al., 2023). Inhibitors of oxidative phosphorylation suppress cuproptosis by inhibiting protein stress response. Inhibitors of mitochondrial pyruvate uptake (UK5099) and blockers of electron transfer chain complexes (rotenone and marital) can attenuate cuproptosis (Hinoi et al., 2006; Simeone et al., 2009).

The discovery of cuproptosis mechanisms has provided a new direction for drug research in future cancer treatments. Drugs related to cuproptosis, such as Cu ionophores that can induce cuproptosis, have a promising application prospect in future cancer treatment (Oliveri, 2020).

### 5.1 Cu ionophores-mediated cancer therapy

**FIGURE 5:**
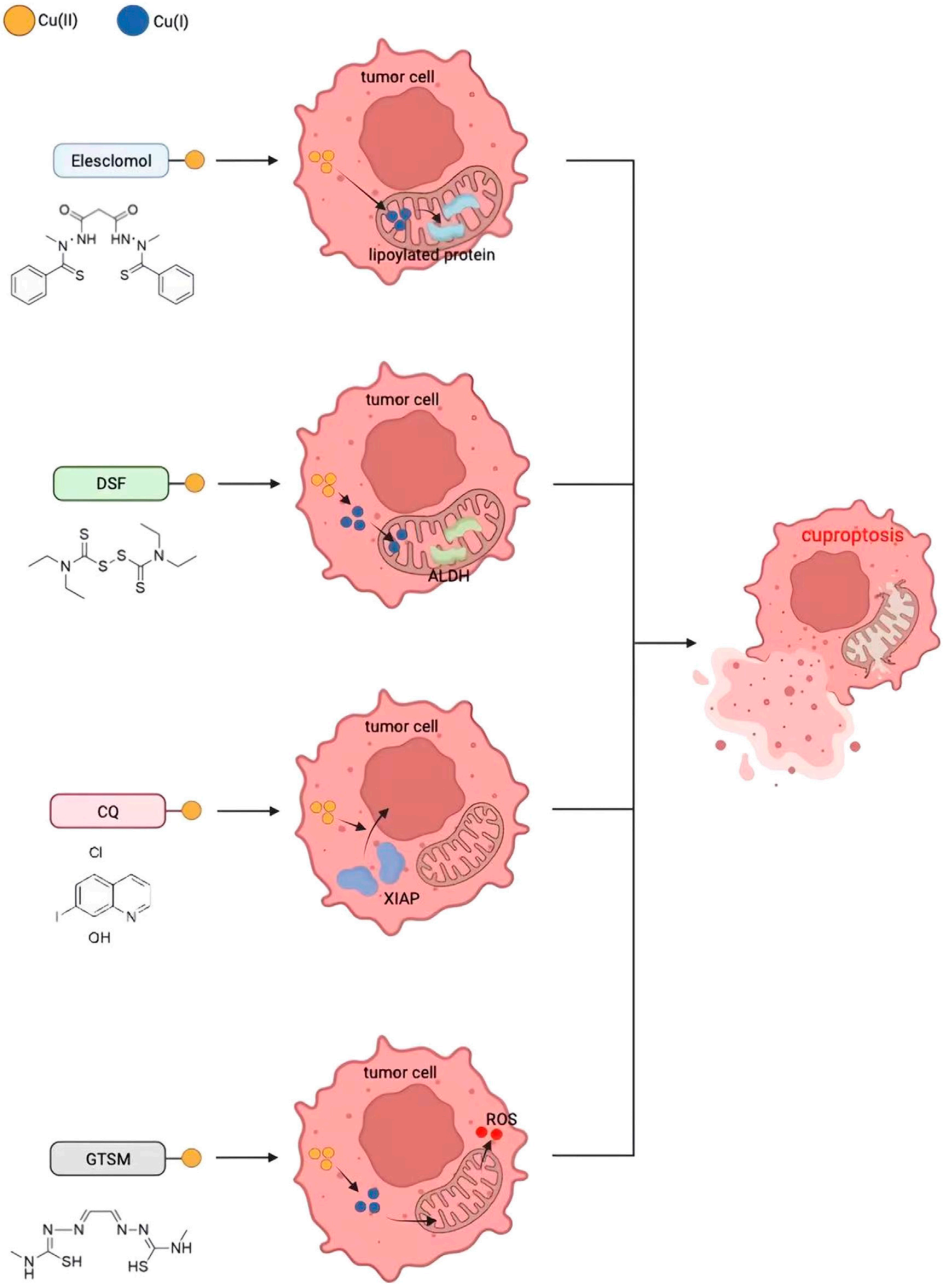
Four potential cancer treatment methods based on cuproptosis.

As described above, Cu ionophores such as ES and DSF can transfer Cu ions into cells and mitochondria, inducing cell death by causing DLAT oligomerization and loss of Fe-S protein clusters. DSF, ES, clioquinol, and bis(thiosemicarbazone) ligands are four commonly used structurally different copper ion carriers whose anti-tumor activity is completely correlated with Cu levels, and the activity of the individual ligands can be ignored (Figure 5) (Cater et al., 2013).

#### 5.1.1 Disulfiram

DSF is an aldehyde dehydrogenase (ALDH) inhibitor that was first approved by the DFA in 1951 for the treatment of alcoholism. Later, due to its affordability, high availability, safety, and anticancer activity, it has become a widely studied anticancer drug (Jiao et al., 2016; Lu et al., 2022; Oliveri, 2022). However, it has the disadvantage of depending on individualized administration of Cu (II) and having limited selective targeting ability (Guan et al., 2023). To overcome this limitation, researchers have combined DSF with Cu (I I) to prepare a mixture of DSF and Cu (DSF-Cu), demonstrating enormous potential for cancer treatment. For example, Allensworth et al. used an inflammatory breast cancer (IBC) model and found that DSF can promote intracellular Cu accumulation independently of the Cu transporter Ctr1. DSF-Cu activated pro-apoptotic redox reactions by inhibiting NF-κB signaling and reducing cellular antioxidant levels. DSF-Cu induced cell apoptosis only in tumor cells and significantly inhibited tumor growth *in vivo* (Allensworth et al., 2015). Similarly, Xueying Ren et al. found that DSF-Cu severely damaged mitochondrial morphology, impaired energy metabolism, accelerated the production of a large amount of ROS, induced DNA double-strand breaks, and accelerated ferroptosis in HCC cell experimental models. DSF-Cu effectively prevented liver cancer cell migration, invasion, and angiogenesis (Ren et al., 2021). In addition, Bing Xu et al. found that DSF-Cu selectively targeted leukemia stem cell-like cells *in vitro* and *in vivo* by activating the ROS-mediated stress-related JNK pathway (e.g., phosphorylation of JNK and c-Jun), while inhibiting the NF-E2-related factor 2 (NRF2) and NF-κB cascade reactions that defend against oxidative stress to induce cell toxicity, inhibit cell proliferation, and induce cell death (Xu et al., 2017). Hassani et al. also found that DSF-Cu induced cell cycle arrest and cell death in acute myeloid leukemia cells by reducing the expression of the oncogene MYC, increasing the expression of tumor suppressor FOXO and the anti-cancer gene PTEN, and disrupting ROS homeostasis (Hassani et al., 2018). Additionally, DSF/Cu(II) induces apoptosis and inhibits cell proliferation in nasopharyngeal carcinoma (NPC) cells by increasing the expression of chloride channel 3 (CIC-3) protein and opening CIC-3 channels (Xu et al., 2019). Subsequently, Yiqiu Li et al. found in their study on NPC that DSF-Cu promoted cell apoptosis and necrosis by increasing cellular ROS levels and activating the MAPK pathway associated with cell apoptosis. DSF-Cu also suppressed the expression of α-smooth muscle actin (α-SMA) and induced apoptosis of cancer-associated fibroblasts (CAFs) to exert its anti-tumor effect (Li et al., 2020).

#### 5.1.2 ES

ES is a well-known Cu ionophore that was discovered through high-throughput screening of compound libraries and a structure-activity relationship (SAR) study targeting human sarcoma cell lines (Babak and Ahn, 2021). The main mechanism of action for ES is thought to involve the initiation of oxidative stress, ultimately triggering apoptosis in the cancer cells (Oliveri, 2022), although other studies have found that ES can cause DNA damage and cell cycle arrest (Hasinoff et al., 2015), as well as induce ferroptosis (Gao et al., 2021). The recent discovery of cuproptosis may provide a novel and plausible explanation for the anticancer mechanism of ES (71).

While the precise anticancer mechanism of ES remains incompletely understood, it is established that the presence of Cu ions in the cellular environment is crucial for its anticancer effect. Outside of cells, ES can form complexes with Cu (II) in serum (Wu et al., 2011). Subsequently, ES-Cu (II) shuttles inside and outside cells, delivering Cu (II) into cells. It is worth noting that, unlike other Cu ionophores such as DSF, after treatment with ES, cellular Cu selectively accumulates in mitochondria, where Cu (II) is reduced to Cu (I) to induce ROS production for anticancer activity. At the same concentration, ES induces a greater increase in cellular Cu than DSF (12). Additionally, it has been reported that the use of ES can degrade Cu transporter ATPase 1 (ATP7A) in colon cancer cells, which is a protein responsible for facilitating the intracellular Cu efflux (Fukai et al., 2018), and the degradation of ATP7A by ES results in the accumulation of Cu ions within the mitochondria of cancer cells (Gao et al., 2021).

The cytotoxicity of ES is attributed to the accumulation of Cu ions in mitochondria, and as early as 2012, researchers discovered that the cytotoxic effect of ES on MDA-MB435 melanoma cells was entirely lost when the cells were cultured in serum-free medium (the only source of Cu in the culture medium) (Nagai et al., 2012). Adding Cu to serum-free medium could rescue the anticancer effect of disulfiram, while other metal ions did not help to rescue the effect of ES (12). Recent reports on copper apoptosis have consistently yielded experimental results in mononuclear cells and lung cancer NCIH2030 cells (Tsvetkov et al., 2022). Therefore, it can be concluded that the cytotoxic effect of ES on cancer cells primarily occurs through the action of Cu ions (Zheng et al., 2022).

#### 5.1.3 HQs

The most renowned compound in HQ is 7-iodo-5-chloro-8-hydroxyquinoline (CQ), which was initially utilized as an antibiotic and has more recently been investigated for its reutilization in various diseases including neurodegenerative disorders and cancer (Oliveri, 2020). Through studies on prostate cancer, it has been established that chloroquine-induced cell death in the cytoplasm is mediated by the migration of X-linked inhibitor of apoptosis protein (XIAP), a cysteine protease activity regulator, to the nucleus, allowing for caspase-dependent cell death, and its cytotoxicity increases with increased copper levels (Cater and Haupt, 2011). The selective properties of Cu-CQ are evident in its ability to induce XIAP clearance exclusively in prostate cancer cells, while having no such effect on normal prostate epithelial cells (Cater and Haupt, 2011). Although CQ exhibits selectivity against cancer cells, it presents serious side effects and may lead to acute myelocytic neuropathy (SMON) (Mao and Schimmer, 2008). Therefore, researchers have also explored alternative derivatives of HQ that may offer better anticancer effects and mitigate side effects. For example, (2-(dimethylamino)methyl-5,7-dichloro-8-hydroxyquinoline) (PBT2) exhibits stronger cell toxicity than CQ in the presence of Cu, and this higher effect of inducing cancer cell death may be attributed to distinct mechanisms of action and alterations induced cellular Cu distribution (Summers et al., 2020).

#### 5.1.4 Bis(thiosemicarbazone) ligands

Bis(thiosemicarbazones) ligands, such as diacetyl-bis [N (4)-methylthiosemicarbazone] Cu^II^ [Cu^II^ (ATSM)] and glyoxal-bis [N (4)-methylthiosemicarbazone] Cu^II^ [Cu^II^ (GTSM)] (Palanimuthu et al., 2013), have been proven to be effective anticancer drugs.

Cu-ATSM and Cu-GTSM have been investigated as potential anticancer agents for both *in vitro* and *in vivo* studies involving prostate cancer cells (Voli et al., 2020). Notably, *in vitro* experiments have demonstrated that Cu-GTSM exhibits greater efficacy than Cu-ATSM in inducing cell death in cancerous prostate PC3 cells. Cu-ATSM and Cu-GTSM have been studied as potential anticancer agents for both *in vitro* and *in vivo* prostate cancer cells (Cater et al., 2013), and *in vitro* experiments have demonstrated that Cu-GTSM is more effective than Cu-ATSM in inducing cell death in cancerous prostate PC3 cells. Additionally, the application of physiological concentrations of Cu to the culture medium notably increases the activity of Cu-GTSM, and Cater et al. demonstrated that Cu-GTSM can effectively utilize extracellular Cu, leading to an elevation of intracellular Cu levels through a metal-responsive element luciferase reporter gene, whereas Cu-ATSM cannot (Cater et al., 2013). This differential behavior is attributed to Cu-ATSM’s inability to release Cu in a reducing environment, whereas GTSM can cause Cu to accumulate inside cells, transport ions across membranes, and release them (Oliveri, 2022).

Further studies on the target of Cu-GTSM have found that, like other Cu ion carriers, the selectivity of Cu-GTSM is associated with the ROS sensitivity of cancer cells. Notably, TRAMP cells (TRAMP-C1) have high levels of ROS and reduced glutathione (reduced GSH), rendering them susceptible to the effect of copper ion carriers (Denoyer et al., 2016).

### 5.2 Nanotechnology-mediated cancer therapy via the cuproptosis pathway

**FIGURE 6:**
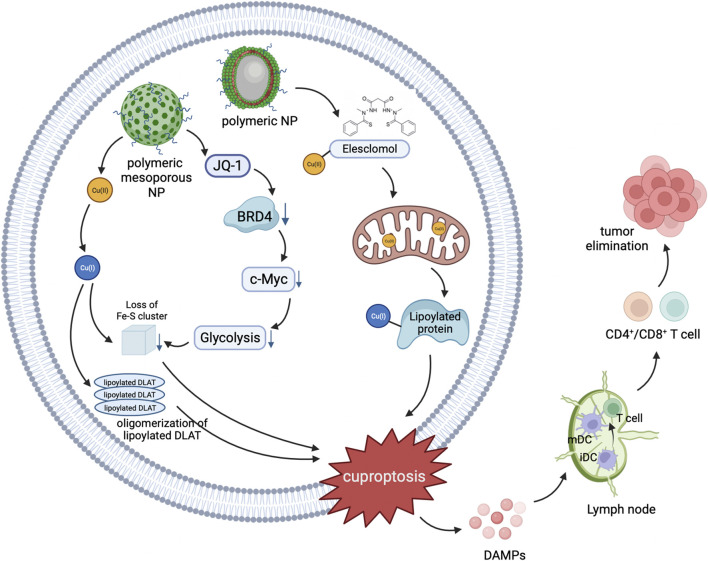
Schematic illustration of nanomedicines mediated cuproptosis cascade with antitumor immunity.

Although cuproptosis has shown promising prospects in cancer therapy, there are still some challenges to overcome. For example, how to selectively increase the Cu concentration in cancer cells, and how to precisely target cancer cells to avoid damaging normal cells (Wei and Fu, 2023). Nanomaterials, due to their unique physicochemical properties and biological effects (Zhong et al., 2022), have the potential to address these issues and are currently widely applied in basic research for cancer diagnosis and treatment, with the aim of achieving clinical applications (Fu et al., 2021). Copper-based nanomaterials can serve as a novel type of cuproptosis inducers, which can achieve active targeting through surface modification, and passively accumulate a large amount of copper at the tumor site through the enhanced permeability and retention (EPR) effect (Figure 6). This ultimately leads to cuproptosis in cancer cells, exerting a therapeutic effect (Wei and Fu, 2023).

Cu (I) plays an important role in cuproptosis-mediated cancer therapy. However, due to its instability, it is difficult to deliver Cu (I) directly into cells, which promotes the transfer of Cu (II) because Cu (II) can be reduced to Cu (I) inside the cell to induce Cu-mediated cell death (Wei and Fu, 2023). Therefore, Zhou et al. developed a DSF-loaded Cu-doped Au@MSN nanoplatform (Au@MSN-Cu/PEG/DSF), with the aim of optimizing therapeutic efficacy while minimizing potential adverse effects on normal cells. After intravenous injection, Au@MSN-Cu/PEG/DSF can accumulate within the tumor region via the EPR effect. After entering cancer cells, it can release a sufficient amount of DSF and Cu (II) through local photothermal-triggered degradation of copper-doped silica framework under near-infrared laser irradiation. The released DSF and Cu (II) react *in situ* to generate cytotoxic DSF-Cu (II), while simultaneously converting Cu (II) to the more toxic Cu (I). *In vitro* and *in vivo* experiments have shown that Au@MSN-Cu/PEG/DSF can effectively hinder tumor growth, and the damage to normal tissues can be negligible with synergistic photothermal therapy (Zhou et al., 2023).

In recent years, nanomaterials used as drug carriers have been endowed with stimulus responsive properties, which can respond to internal or external stimuli such as temperature, pH, redox reactions, etc. Thus, they can release drugs in a controllable kinetics at specific locations, improving the efficiency of cancer drug therapy while protecting non-targeted tissues (Hajebi et al., 2019). For instance, Jia et al., 2023 designed a brain-targeted nanoplatform (HFn-Cu-REGO NPs) by incorporating human H-ferritin (HFn), regorafenib (an orally bioavailable multikinase inhibitor that significantly inhibits glioblastoma multiforme (GBM)), and Cu (II). The Cu (II)-mediated cuproptosis, together with the autophagy arrest mediated by regorafenib via the suppression of autophagosome-lysosome formation (Jiang et al., 2020), can effectively inhibit GBM. Moreover, with the added ability to precisely target cancer cells by binding to transferrin receptor 1 (TfR1) ability (Song et al., 2021), pH-responsive delivery, and blood-brain barrier (BBB) penetration capability (Fan et al., 2018) endowed by HFn, regorafenib and Cu(II) can be accurately delivered to cancer cells. This allows HFn-Cu-REGO NPs to exhibit excellent GBM inhibition without causing harm to healthy tissues.

Furthermore, there are many studies utilizing nanosystems based on cuproptosis mechanism for cancer therapy, which have promoted the development of Cu-based nanomaterials as inducers of cuproptosis for eradicating malignant tumors. Unfortunately, our understanding of the complex molecular mechanisms and regulatory pathways of copper-induced cell death is still limited. Further understanding of the cuproptosis mechanism, as well as a deeper understanding of the relationship between cuproptosis and nanomaterials, will facilitate the development of more effective anti-cancer nanomaterials.

## 6 Conclusion and perspectives

Cu is an essential element in living organisms, and is typically maintained at minimal levels within mammalian cells. However, when the concentration of Cu ions in cells exceeds the threshold for maintaining homeostasis, it exhibits cytotoxicity. Many studies have observed a strong correlation between disease status and Cu, with various types of malignant tumors showing higher levels of Cu compared to normal tissue, and Cu playing a critical role in the occurrence, severity, and progression of cancer. Therefore, it has been suggested that altering Cu levels may be a promising approach for preventing cancer development and achieving therapeutic effects. A series of related studies have been conducted, including research on Cu ion carriers for anti-cancer therapy, which have discovered a form of cell death mediated by Cu. This was subsequently named cuproptosis and further research has been conducted on its mechanism and application in cancer treatment.

This article reviews the discovery, naming, research, and detailed mechanism of cuproptosis and its application in cancer treatment. It describes the latest understanding of the cuproptosis mechanism and the recent anti-cancer treatment methods based on this mechanism. However, despite the extensive research on the cuproptosis mechanism, there are still some questions to be answered, such as whether there are other pathways involved in cuproptosis besides protein lipidation, whether there are biomarkers that can predict cuproptosis occurrence, and what is the optimal range of Cu ion concentration for achieving the best therapeutic effect without damaging normal cells. How to control Cu ion concentration effectively and how to choose the appropriate drug type, dosage, and timing for patients with tumor heterogeneity are also important issues to be addressed.

In conclusion, the discovery and application of cuproptosis provide a new direction for cancer treatment. With further research, we will have a deeper understanding of the mechanism of cuproptosis and develop more effective cancer treatment strategies based on this mechanism.

## References

[B1] Allensworth J. L. Evans M. K. Bertucci F. Aldrich A. J. Festa R. A. Finetti P. (2015). Disulfiram (DSF) acts as a copper ionophore to induce copper-dependent oxidative stress and mediate anti-tumor efficacy in inflammatory breast cancer. Mol. Oncol. 9 (6), 1155–1168. 10.1016/j.molonc.2015.02.007 25769405 PMC4493866

[B2] Aubert L. Nandagopal N. Steinhart Z. Lavoie G. Nourreddine S. Berman J. (2020). Copper bioavailability is a KRAS-specific vulnerability in colorectal cancer. Nat. Commun. 11 (1), 3701. 10.1038/s41467-020-17549-y 32709883 PMC7381612

[B3] Babak M. V. Ahn D. (2021). Modulation of intracellular copper levels as the mechanism of action of anticancer copper complexes: clinical relevance. Biomedicines 9 (8), 852. 10.3390/biomedicines9080852 34440056 PMC8389626

[B4] Baltaci A. K. Dundar T. K. Aksoy F. Mogulkoc R. (2017). Changes in the serum levels of trace elements before and after the operation in thyroid cancer patients. Biol. Trace Elem. Res. 175 (1), 57–64. 10.1007/s12011-016-0768-2 27263537

[B5] Brady D. C. Crowe M. S. Turski M. L. Hobbs G. A. Yao X. Chaikuad A. (2014). Copper is required for oncogenic BRAF signalling and tumorigenesis. Nature 509 (7501), 492–496. 10.1038/nature13180 24717435 PMC4138975

[B6] Buccarelli M. D'Alessandris Q. G. Matarrese P. Mollinari C. Signore M. Cappannini A. (2021). Elesclomol-induced increase of mitochondrial reactive oxygen species impairs glioblastoma stem-like cell survival and tumor growth. J. Exp. Clin. Cancer Res. 40 (1), 228. 10.1186/s13046-021-02031-4 34253243 PMC8273992

[B7] Cater M. A. Haupt Y. (2011). Clioquinol induces cytoplasmic clearance of the X-linked inhibitor of apoptosis protein (XIAP): therapeutic indication for prostate cancer. Biochem. J. 436 (2), 481–491. 10.1042/BJ20110123 21426304

[B8] Cater M. A. Pearson H. B. Wolyniec K. Klaver P. Bilandzic M. Paterson B. M. (2013). Increasing intracellular bioavailable copper selectively targets prostate cancer cells. ACS Chem. Biol. 8 (7), 1621–1631. 10.1021/cb400198p 23656859

[B9] Chillappagari S. Seubert A. Trip H. Kuipers O. P. Marahiel M. A. Miethke M. (2010). Copper stress affects iron homeostasis by destabilizing iron-sulfur cluster formation in Bacillus subtilis. J. Bacteriol. 192 (10), 2512–2524. 10.1128/JB.00058-10 20233928 PMC2863568

[B10] De Luca A. Barile A. Arciello M. Rossi L. (2019). Copper homeostasis as target of both consolidated and innovative strategies of anti-tumor therapy. J. Trace Elem. Med. Biol. 55, 204–213. 10.1016/j.jtemb.2019.06.008 31345360

[B11] Denoyer D. Masaldan S. La Fontaine S. Cater M. A. (2015). Targeting copper in cancer therapy: 'Copper that Cancer. Metallomics 7 (11), 1459–1476. 10.1039/c5mt00149h 26313539

[B12] Denoyer D. Pearson H. B. Clatworthy S. A. Smith Z. M. Francis P. S. Llanos R. M. (2016). Copper as a target for prostate cancer therapeutics: copper-ionophore pharmacology and altering systemic copper distribution. Oncotarget 7 (24), 37064–37080. 10.18632/oncotarget.9245 27175597 PMC5095059

[B13] Elmore S. (2007). Apoptosis: a review of programmed cell death. Toxicol. Pathol. 35 (4), 495–516. 10.1080/01926230701320337 17562483 PMC2117903

[B14] Fan K. Jia X. Zhou M. Wang K. Conde J. He J. (2018). Ferritin nanocarrier traverses the blood brain barrier and kills glioma. ACS Nano 12 (5), 4105–4115. 10.1021/acsnano.7b06969 29608290

[B15] Festa R. A. Thiele D. J. (2011). Copper: an essential metal in biology. Curr. Biol. 21 (21), R877–R883. 10.1016/j.cub.2011.09.040 22075424 PMC3718004

[B16] Fu Q. Zhang X. Song J. Yang H. (2021). Plasmonic gold nanoagents for cancer imaging and therapy. VIEW 2 (5), 20200149. 10.1002/viw.20200149

[B17] Fukai T. Ushio-Fukai M. Kaplan J. H. (2018). Copper transporters and copper chaperones: roles in cardiovascular physiology and disease. Am. J. Physiol. Cell. Physiol. 315 (2), C186–c201. 10.1152/ajpcell.00132.2018 29874110 PMC6139499

[B18] Gao W. Huang Z. Duan J. Nice E. C. Lin J. Huang C. (2021). Elesclomol induces copper-dependent ferroptosis in colorectal cancer cells via degradation of ATP7A. Mol. Oncol. 15 (12), 3527–3544. 10.1002/1878-0261.13079 34390123 PMC8637554

[B19] Gao X. Huang H. Pan C. Mei Z. Yin S. Zhou L. (2022). Disulfiram/copper induces immunogenic cell death and enhances CD47 blockade in hepatocellular carcinoma. Cancers (Basel) 14 (19), 4715. 10.3390/cancers14194715 36230638 PMC9564202

[B20] Gérard C. Bordeleau L. J. Barralet J. Doillon C. J. (2010). The stimulation of angiogenesis and collagen deposition by copper. Biomaterials 31 (5), 824–831. 10.1016/j.biomaterials.2009.10.009 19854506

[B21] Guan D. Zhao L. Shi X. Ma X. Chen Z. (2023). Copper in cancer: from pathogenesis to therapy. Biomed. Pharmacother. 163, 114791. 10.1016/j.biopha.2023.114791 37105071

[B22] Guo J. Cheng J. Zheng N. Zhang X. Dai X. Zhang L. (2021). Copper promotes tumorigenesis by activating the PDK1-AKT oncogenic pathway in a copper transporter 1 dependent manner. Adv. Sci. (Weinh). 8 (18), e2004303. 10.1002/advs.202004303 34278744 PMC8456201

[B23] Hajebi S. Rabiee N. Bagherzadeh M. Ahmadi S. Rabiee M. Roghani-Mamaqani H. (2019). Stimulus-responsive polymeric nanogels as smart drug delivery systems. Acta Biomater. 92, 1–18. 10.1016/j.actbio.2019.05.018 31096042 PMC6661071

[B24] Halliwell B. Gutteridge J. M. (1984). Oxygen toxicity, oxygen radicals, transition metals and disease. Biochem. J. 219 (1), 1–14. 10.1042/bj2190001 6326753 PMC1153442

[B25] Hasinoff B. B. Wu X. Yadav A. A. Patel D. Zhang H. Wang D. S. (2015). Cellular mechanisms of the cytotoxicity of the anticancer drug elesclomol and its complex with Cu(II). Biochem. Pharmacol. 93 (3), 266–276. 10.1016/j.bcp.2014.12.008 25550273

[B26] Hassani S. Ghaffari P. Chahardouli B. Alimoghaddam K. Ghavamzadeh A. Alizadeh S. (2018). Disulfiram/copper causes ROS levels alteration, cell cycle inhibition, and apoptosis in acute myeloid leukaemia cell lines with modulation in the expression of related genes. Biomed. Pharmacother. 99, 561–569. 10.1016/j.biopha.2018.01.109 29902866

[B27] He F. Chang C. Liu B. Li Z. Li H. Cai N. (2019). Copper (II) ions activate ligand-independent receptor tyrosine kinase (RTK) signaling pathway. BioMed Res. Int. 2019, 4158415. 10.1155/2019/4158415 31218225 PMC6537018

[B28] Hinoi E. Takarada T. Tsuchihashi Y. Fujimori S. Moriguchi N. Wang L. (2006). A molecular mechanism of pyruvate protection against cytotoxicity of reactive oxygen species in osteoblasts. Mol. Pharmacol. 70 (3), 925–935. 10.1124/mol.106.024398 16766717

[B29] Holohan C. Van Schaeybroeck S. Longley D. B. Johnston P. G. (2013). Cancer drug resistance: an evolving paradigm. Nat. Rev. Cancer 13 (10), 714–726. 10.1038/nrc3599 24060863

[B30] Jia W. Tian H. Jiang J. Zhou L. Li L. Luo M. (2023). Brain-targeted HFn-Cu-rego nanoplatform for site-specific delivery and manipulation of autophagy and cuproptosis in glioblastoma. Small 19 (2), e2205354. 10.1002/smll.202205354 36399643

[B31] Jiang A. Luo P. Chen M. Fang Y. Liu B. Wu Z. (2022). A new thinking: deciphering the aberrance and clinical implication of copper-death signatures in clear cell renal cell carcinoma. Cell. Biosci. 12 (1), 209. 10.1186/s13578-022-00948-7 36581992 PMC9801655

[B32] Jiang J. Zhang L. Chen H. Lei Y. Zhang T. Wang Y. (2020). Regorafenib induces lethal autophagy arrest by stabilizing PSAT1 in glioblastoma. Autophagy 16 (1), 106–122. 10.1080/15548627.2019.1598752 30909789 PMC6984601

[B33] Jiao Y. Hannafon B. N. Ding W. Q. (2016). Disulfiram's anticancer activity: evidence and mechanisms. Anticancer Agents Med. Chem. 16 (11), 1378–1384. 10.2174/1871520615666160504095040 27141876

[B34] Jin Y. Zhang C. Xu H. Xue S. Wang Y. Hou Y. (2011). Combined effects of serum trace metals and polymorphisms of CYP1A1 or GSTM1 on non-small cell lung cancer: a hospital based case-control study in China. Cancer Epidemiol. 35 (2), 182–187. 10.1016/j.canep.2010.06.004 20638923

[B35] Lener M. R. Scott R. J. Wiechowska-Kozłowska A. Serrano-Fernández P. Baszuk P. Jaworska-Bieniek K. (2016). Serum concentrations of selenium and copper in patients diagnosed with pancreatic cancer. Cancer Res. Treat. 48 (3), 1056–1064. 10.4143/crt.2015.282 26727715 PMC4946347

[B36] Li Y. (2020). Copper homeostasis: emerging target for cancer treatment. IUBMB Life 72 (9), 1900–1908. 10.1002/iub.2341 32599675

[B37] Li Y. Chen F. Chen J. Chan S. He Y. Liu W. (2020). Disulfiram/copper induces antitumor activity against both nasopharyngeal cancer cells and cancer-associated fibroblasts through ROS/MAPK and ferroptosis pathways. Cancers (Basel) 12 (1), 138. 10.3390/cancers12010138 31935835 PMC7017005

[B38] Li Y. Q. Chen J. Yin J. Y. Liu Z. Q. Li X. P. (2018). Gene expression and single nucleotide polymorphism of ATP7B are associated with platinum-based chemotherapy response in non-small cell lung cancer patients. J. Cancer 9 (19), 3532–3539. 10.7150/jca.26286 30310510 PMC6171024

[B39] Liu W. Q. Lin W. R. Yan L. Xu W. H. Yang J. (2023). Copper homeostasis and cuproptosis in cancer immunity and therapy. Immunol. Rev. 10.1111/imr.13276 37715546

[B40] Lu Y. Pan Q. Gao W. Pu Y. Luo K. He B. (2022). Leveraging disulfiram to treat cancer: mechanisms of action, delivery strategies, and treatment regimens. Biomaterials 281, 121335. 10.1016/j.biomaterials.2021.121335 34979419

[B41] Macomber L. Imlay J. A. (2009). The iron-sulfur clusters of dehydratases are primary intracellular targets of copper toxicity. Proc. Natl. Acad. Sci. U. S. A. 106 (20), 8344–8349. 10.1073/pnas.0812808106 19416816 PMC2688863

[B42] Mao X. Schimmer A. D. (2008). The toxicology of Clioquinol. Toxicol. Lett. 182 (1-3), 1–6. 10.1016/j.toxlet.2008.08.015 18812216

[B43] Matarrese P. Tinari A. Mormone E. Bianco G. A. Toscano M. A. Ascione B. (2005). Galectin-1 sensitizes resting human T lymphocytes to Fas (CD95)-mediated cell death via mitochondrial hyperpolarization, budding, and fission. J. Biol. Chem. 280 (8), 6969–6985. 10.1074/jbc.M409752200 15556941

[B44] Maung M. T. Carlson A. Olea-Flores M. Elkhadragy L. Schachtschneider K. M. Navarro-Tito N. (2021). The molecular and cellular basis of copper dysregulation and its relationship with human pathologies. Faseb J. 35 (9), e21810. 10.1096/fj.202100273RR 34390520

[B45] Mayr J. A. Feichtinger R. G. Tort F. Ribes A. Sperl W. (2014). Lipoic acid biosynthesis defects. J. Inherit. Metab. Dis. 37 (4), 553–563. 10.1007/s10545-014-9705-8 24777537

[B46] Migocka M. (2015). Copper-transporting ATPases: the evolutionarily conserved machineries for balancing copper in living systems. IUBMB Life 67 (10), 737–745. 10.1002/iub.1437 26422816

[B47] Mun E. J. Babiker H. M. Weinberg U. Kirson E. D. Von Hoff D. D. (2018). Tumor-treating fields: a fourth modality in cancer treatment. Clin. Cancer Res. 24 (2), 266–275. 10.1158/1078-0432.CCR-17-1117 28765323

[B48] Nagai M. Vo N. H. Shin Ogawa L. Chimmanamada D. Inoue T. Chu J. (2012). The oncology drug elesclomol selectively transports copper to the mitochondria to induce oxidative stress in cancer cells. Free Radic. Biol. Med. 52 (10), 2142–2150. 10.1016/j.freeradbiomed.2012.03.017 22542443

[B49] Nowell C. S. Radtke F. (2017). Notch as a tumour suppressor. Nat. Rev. Cancer 17 (3), 145–159. 10.1038/nrc.2016.145 28154375

[B50] Oliveri V. (2020). Biomedical applications of copper ionophores. Coord. Chem. Rev. 422, 213474. 10.1016/j.ccr.2020.213474

[B51] Oliveri V. (2022). Selective targeting of cancer cells by copper ionophores: an overview. Front. Mol. Biosci. 9, 841814. 10.3389/fmolb.2022.841814 35309510 PMC8931543

[B52] Ostrakhovitch E. A. Lordnejad M. R. Schliess F. Sies H. Klotz L.-O. (2002). Copper ions strongly activate the phosphoinositide-3-kinase/akt pathway independent of the generation of reactive oxygen species. Archives Biochem. Biophysics 397 (2), 232–239. 10.1006/abbi.2001.2559 11795876

[B53] Palanimuthu D. Shinde S. V. Somasundaram K. Samuelson A. G. (2013). *In vitro* and *in vivo* anticancer activity of copper bis(thiosemicarbazone) complexes. J. Med. Chem. 56 (3), 722–734. 10.1021/jm300938r 23320568

[B54] Parisi L. R. Morrow L. M. Visser M. B. Atilla-Gokcumen G. E. (2018). Turning the spotlight on lipids in non-apoptotic cell death. ACS Chem. Biol. 13 (3), 506–515. 10.1021/acschembio.7b01082 29376324

[B55] Parr-Sturgess C. A. Tinker C. L. Hart C. A. Brown M. D. Clarke N. W. Parkin E. T. (2012). Copper modulates zinc metalloproteinase-dependent ectodomain shedding of key signaling and adhesion proteins and promotes the invasion of prostate cancer epithelial cells. Mol. Cancer Res. 10 (10), 1282–1293. 10.1158/1541-7786.MCR-12-0312 22936788

[B56] Pavithra V. Sathisha T. G. Kasturi K. Mallika D. S. Amos S. J. Ragunatha S. (2015). Serum levels of metal ions in female patients with breast cancer. J. Clin. Diagn Res. 9 (1), BC25–Bc27. 10.7860/JCDR/2015/11627.5476 25737978 PMC4347069

[B57] Ren X. Li Y. Zhou Y. Hu W. Yang C. Jing Q. (2021). Overcoming the compensatory elevation of NRF2 renders hepatocellular carcinoma cells more vulnerable to disulfiram/copper-induced ferroptosis. Redox Biol. 46, 102122. 10.1016/j.redox.2021.102122 34482117 PMC8416961

[B58] Sabharwal S. S. Schumacker P. T. (2014). Mitochondrial ROS in cancer: initiators, amplifiers or an Achilles' heel? Nat. Rev. Cancer 14 (11), 709–721. 10.1038/nrc3803 25342630 PMC4657553

[B59] Saleh S. A. K. Adly H. M. Abdelkhaliq A. A. Nassir A. M. (2020). Serum levels of selenium, zinc, copper, manganese, and iron in prostate cancer patients. Curr. Urol. 14 (1), 44–49. 10.1159/000499261 32398996 PMC7206590

[B60] Sethi N. Kang Y. (2011). Notch signalling in cancer progression and bone metastasis. Br. J. Cancer 105 (12), 1805–1810. 10.1038/bjc.2011.497 22075946 PMC3251892

[B61] Shen S. Zhang Z. Huang H. Yang J. Tao X. Meng Z. (2023). Copper-induced injectable hydrogel with nitric oxide for enhanced immunotherapy by amplifying immunogenic cell death and regulating cancer associated fibroblasts. Biomater. Res. 27 (1), 44. 10.1186/s40824-023-00389-4 37165428 PMC10170699

[B62] Simeone V. Baser N. Perrelli D. Cesari G. El Bilali H. Natale P. (2009). Residues of rotenone, azadirachtin, pyrethrins and copper used to control Bactrocera oleae (Gmel.) in organic olives and oil. Food Addit. Contam. Part A Chem. Anal. Control Expo. Risk Assess. 26 (4), 475–481. 10.1080/02652030802562938 19680921

[B63] Solier S. Müller S. Cañeque T. Versini A. Mansart A. Sindikubwabo F. (2023). A druggable copper-signalling pathway that drives inflammation. Nature 617 (7960), 386–394. 10.1038/s41586-023-06017-4 37100912 PMC10131557

[B64] Solmonson A. DeBerardinis R. J. (2018). Lipoic acid metabolism and mitochondrial redox regulation. J. Biol. Chem. 293 (20), 7522–7530. 10.1074/jbc.TM117.000259 29191830 PMC5961061

[B65] Song N. Zhang J. Zhai J. Hong J. Yuan C. Ferritin L. M. (2021). Ferritin: a multifunctional nanoplatform for biological detection, imaging diagnosis, and drug delivery. Acc. Chem. Res. 54 (17), 3313–3325. 10.1021/acs.accounts.1c00267 34415728

[B66] Summers K. L. Dolgova N. V. Gagnon K. B. Sopasis G. J. James A. K. Lai B. (2020). PBT2 acts through a different mechanism of action than other 8-hydroxyquinolines: an X-ray fluorescence imaging study. Metallomics 12 (12), 1979–1994. 10.1039/d0mt00222d 33169753

[B67] Sung H. Ferlay J. Siegel R. L. Laversanne M. Soerjomataram I. Jemal A. (2021). Global cancer statistics 2020: GLOBOCAN estimates of incidence and mortality worldwide for 36 cancers in 185 countries. CA Cancer J. Clin. 71 (3), 209–249. 10.3322/caac.21660 33538338

[B68] Tisato F. Marzano C. Porchia M. Pellei M. Santini C. (2010). Copper in diseases and treatments, and copper-based anticancer strategies. Med. Res. Rev. 30 (4), 708–749. 10.1002/med.20174 19626597

[B69] Topalian S. L. Hodi F. S. Brahmer J. R. Gettinger S. N. Smith D. C. McDermott D. F. (2012). Safety, activity, and immune correlates of anti-PD-1 antibody in cancer. N. Engl. J. Med. 366 (26), 2443–2454. 10.1056/NEJMoa1200690 22658127 PMC3544539

[B70] Tsang T. Posimo J. M. Gudiel A. A. Cicchini M. Feldser D. M. Brady D. C. (2020). Copper is an essential regulator of the autophagic kinases ULK1/2 to drive lung adenocarcinoma. Nat. Cell. Biol. 22 (4), 412–424. 10.1038/s41556-020-0481-4 32203415 PMC7610258

[B71] Tsvetkov P. Coy S. Petrova B. Dreishpoon M. Verma A. Abdusamad M. (2022). Copper induces cell death by targeting lipoylated TCA cycle proteins. Science 375 (6586), 1254–1261. 10.1126/science.abf0529 35298263 PMC9273333

[B72] Tsvetkov P. Detappe A. Cai K. Keys H. R. Brune Z. Ying W. (2019). Mitochondrial metabolism promotes adaptation to proteotoxic stress. Nat. Chem. Biol. 15 (7), 681–689. 10.1038/s41589-019-0291-9 31133756 PMC8183600

[B73] Vallières C. Holland S. L. Avery S. V. (2017). Mitochondrial ferredoxin determines vulnerability of cells to copper excess. Cell. Chem. Biol. 24 (10), 1228–1237. 10.1016/j.chembiol.2017.08.005 28867595 PMC5654725

[B74] Voli F. Valli E. Lerra L. Kimpton K. Saletta F. Giorgi F. M. (2020). Intratumoral copper modulates PD-L1 expression and influences tumor immune evasion. Cancer Res. 80 (19), 4129–4144. 10.1158/0008-5472.CAN-20-0471 32816860

[B75] Wei C. Fu Q. (2023). Cell death mediated by nanotechnology via the cuproptosis pathway: a novel horizon for cancer therapy. VIEW 4 (3), 20230001. 10.1002/viw.20230001

[B76] Wu D. Wang S. Yu G. Chen X. (2021). Cell death mediated by the pyroptosis pathway with the aid of nanotechnology: prospects for cancer therapy. Angew. Chem. Int. Ed. Engl. 60 (15), 8018–8034. 10.1002/anie.202010281 32894628

[B77] Wu L. Zhou L. Liu D. Q. Vogt F. G. Kord A. S. (2011). LC-MS/MS and density functional theory study of copper(II) and nickel(II) chelating complexes of elesclomol (a novel anticancer agent). J. Pharm. Biomed. Anal. 54 (2), 331–336. 10.1016/j.jpba.2010.09.007 20933353

[B78] Xie H. Kang Y. J. (2009). Role of copper in angiogenesis and its medicinal implications. Curr. Med. Chem. 16 (10), 1304–1314. 10.2174/092986709787846622 19355887

[B79] Xie J. Yang Y. Gao Y. He J. (2023). Cuproptosis: mechanisms and links with cancers. Mol. Cancer 22 (1), 46. 10.1186/s12943-023-01732-y 36882769 PMC9990368

[B80] Xu B. Wang S. Li R. Chen K. He L. Deng M. (2017). Disulfiram/copper selectively eradicates AML leukemia stem cells *in vitro* and *in vivo* by simultaneous induction of ROS-JNK and inhibition of NF-κB and Nrf2. Cell. Death Dis. 8 (5), e2797. 10.1038/cddis.2017.176 28518151 PMC5520701

[B81] Xu X. Xu J. Zhao C. Hou X. Li M. Wang L. (2019). Antitumor effects of disulfiram/copper complex in the poorly-differentiated nasopharyngeal carcinoma cells via activating ClC-3 chloride channel. Biomed. Pharmacother. 120, 109529. 10.1016/j.biopha.2019.109529 31606620

[B82] Yadav A. A. Patel D. Wu X. Hasinoff B. B. (2013). Molecular mechanisms of the biological activity of the anticancer drug elesclomol and its complexes with Cu(II), Ni(II) and Pt(II). J. Inorg. Biochem. 126, 1–6. 10.1016/j.jinorgbio.2013.04.013 23707906

[B83] Zheng P. Zhou C. Lu L. Liu B. Ding Y. (2022). Elesclomol: a copper ionophore targeting mitochondrial metabolism for cancer therapy. J. Exp. Clin. Cancer Res. 41 (1), 271. 10.1186/s13046-022-02485-0 36089608 PMC9465867

[B84] Zheng Z. Zhang J. Jiang J. He Y. Zhang W. Mo X. (2020). Remodeling tumor immune microenvironment (TIME) for glioma therapy using multi-targeting liposomal codelivery. J. Immunother. Cancer 8 (2), e000207. 10.1136/jitc-2019-000207 32817393 PMC7437977

[B85] Zhong X. Dai X. Wang Y. Wang H. Qian H.-s. Wang X. (2022). Copper‐based nanomaterials for cancer theranostics. WIREs Nanomedicine Nanobiotechnology 14, 14. 10.1002/wnan.1797 35419993

[B86] Zhou B. Guo L. Zhang B. Liu S. Zhang K. Yan J. (2019). Disulfiram combined with copper induces immunosuppression via PD-L1 stabilization in hepatocellular carcinoma. Am. J. Cancer Res. 9 (11), 2442–2455. 31815045 PMC6895448

[B87] Zhou J. Yu Q. Song J. Li S. Li X.-L. Kang B. (2023). Photothermally triggered copper payload release for cuproptosis-promoted cancer synergistic therapy. Angew. Chem. Int. Ed. 62 (12), e202213922. 10.1002/anie.202213922 36585379

